# Relationship between SRD5A2 rs9282858 polymorphism and the susceptibility of prostate cancer: A meta-analysis based on 20 publications

**DOI:** 10.1097/MD.0000000000006791

**Published:** 2017-05-12

**Authors:** Cheng Fang, Zhong-Qiang Guo, Xiao-Yan Chen, Tong-Zu Liu, Xian-Tao Zeng, Xing-Huan Wang

**Affiliations:** a Center for Evidence-Based and Translational Medicine; b Department of Urology, Zhongnan Hospital of Wuhan University, Wuhan, China.

**Keywords:** meta-analysis, PCa, polymorphism, *SRD5A2*

## Abstract

The pathogenetic mechanism of prostate cancer (PCa) has not been understood completely, and gene polymorphisms have been demonstrated to play a critical role in the course. It has been reported that rs9282858 polymorphism of steroid 5-α-reductase type 2 (*SRD5A2*) may affect the susceptibility of PCa, but some researches showed different results. We therefore carried out a meta-analysis to clarify this relationship.

Relevant studies were identified through PubMed and Chinese National Knowledge Infrastructure databases concerning the association between *SRD5A2* rs9282858 polymorphism and PCa. Odds ratios (ORs) with their 95% confidence intervals (95% CIs) were calculated to assess the strength of the association. Additionally, stratified analyses were performed based on ethnicity and source of control. Besides, heterogeneity test, sensitivity analysis, and publication bias evaluation were conducted in current meta-analysis as well.

Ultimately, 20 publications incorporating 30 case-control studies were included in this meta-analysis, involving a total of 7300 cases and 7952 controls. The overall results demonstrated that *SRD5A2* rs9282858 polymorphism was remarkably associated with increased susceptibility of PCa (TT vs. AA: OR = 4.08, 95% CI = 1.94–8.58; TT + AT vs. AA: OR = 1.28, 95% CI = 1.11–1.47; TT vs. AA + AT: OR = 4.44, 95% CI = 2.12–9.27; allele T vs. allele A: OR = 1.34, 95% CI = 1.17–1.54). After subgroup analyses by ethnicity and source of control, we also observed a similar trend in Latinos, other-ethnicity, population-based, and hospital-based groups under corresponding genetic models.

Our findings indicate that *SRD5A2* rs9282858 polymorphism may be a susceptible factor to PCa.

## 1 Introduction

Prostate cancer (PCa) is the most common malignancy in male reproductive system.^[1]^ In Europe, PCa accounts for 12% of all male cancers, and 9% of tumor-related deaths in adult males.^[2]^ It is estimated that there are approximately 238,590 newly diagnosed PCa patients and 29,720 deaths caused by it in America in 2013.^[3]^ Although the occurrence rate of PCa has been at a low level for a long time in China, it is rising significantly in recent years as a result of the changes in dietary structure and environment, making PCa become the third malignancy of male urogenital system in the country nowadays.^[4]^ It is well known that age and race are two of the most important risk factors to PCa.^[5–6]^ Documents reveal that PCa is rare in men younger than 40 years, but its morbidity increases with age more rapidly than any other cancers in men.^[6]^ As a public security issue all over the world,^[7]^ this malignancy still threatens the health, even life, of aged males in spite of notable improvements in the techniques for the diagnosis and treatment.

Cancer is essentially a genetic disease, and its genesis and development involve a multistep, multistage, and polygenic process.^[8]^ The occurrence and growth of PCa are induced by the changes of multiple genes and stimulation from environmental factors.^[9]^ At present, researchers have paid close attention to genetic association studies about the relationships of cancer with oncogenes, tumor-suppressor genes, and other genes involved in hormonal environment.

Androgen reportedly plays a vital role in the canceration of prostate, so relevant genes involved in biosynthesis and metabolic pathway of androgen may influence the pathogenesis of this malignancy.^[10]^ As is well known, there are 2 main types of androgen in humans and other mammals, namely, testosterone and dihydrotestosterone (DHT).^[10,11]^ Steroid 5α-reductase can restore testosterone into stronger DHT, which has a significant impact on the growth and differentiation of prostate cells.^[11]^ Two types of steroid 5α-reductase have been identified so far, which are steroid 5α-reductase type 1 (SRD5A1) and steroid 5α-reductase type 2 (SRD5A2). Among them, SRD5A2 is highly expressed in androgen-sensitive tissues, such as prostate and testis. In addition, catalyzing SRD5A2 can remarkably enhance the biological activity of DHT and its affinity for androgen receptor (AR), thereby possibly affecting the onset risk of PCa.^[12]^

A large number of researches have been performed to explore the effects of *SRD5A2* polymorphisms on PCa susceptibility during the past decade, but the conclusions remained inconsistent.^[13–15]^ In this study, we searched all eligible publications to carry out a meta-analysis for a clearer perspective on the association of *SRD5A2* rs9282858 polymorphism and the susceptibility of PCa.

## 2 Materials and methods

### 2.1 Literature search

This meta-analysis was conducted in accordance with the checklist of the Meta-analysis of Observational Studies in Epidemiology guidelines. The proposed checklist contains specifications for reporting of meta-analyses of observational studies in epidemiology, including background, search strategy, methods, results, discussion, and conclusions.^[16]^ The PubMed and Chinese National Knowledge Infrastructure databases were searched for all related publications about the association between *SRD5A2* rs9282858 polymorphism and PCa. No language restrictions were imposed on literature search strategy. The keywords applied in searches were “steroid 5α-reductase type 2 or *SRD5A2*” in combination with “prostate cancer” and “polymorphism or mutation or variation.” Moreover, the references of all pertinent articles were manually checked to identify additional relevant studies. All analyses were based on previous published studies; thus, ethical approval was not necessary for this meta-analysis.

### 2.2 Inclusion and exclusion criteria

Studies included in the meta-analysis were required to fulfil the following criteria: they were designed as case-control studies; assessing the correlation between *SRD5A2* rs9282858 polymorphism and PCa susceptibility; presenting adequate genotype data for calculating pooled odds ratios (ORs) and 95% confidence intervals (95% CIs). Apart from articles not meeting the inclusion criteria, reviews, meta-analysis, and repeated publications were excluded.

### 2.3 Data extraction

Data extraction was completed by 2 investigators independently, and all the information was recorded in a standardized form. The results were compared and disagreements were resolved through discussion to reach a consensus. The following data were gathered from each relevant study: the first author's name, year of publication, original country, ethnicity, control source, genotyping method, numbers of cases and controls, genotype frequencies in case and control groups, and *P* values for Hardy-Weinberg equilibrium (HWE) in controls.

### 2.4 Statistical analysis

The genetic relationship between *SRD5A2* rs9282858 polymorphism and PCa susceptibility was examined through crude ORs and 95% CIs under TT vs. AA, TT + AT vs. AA, TT vs. AA + AT, allele T vs. allele A, and AT vs. AA contrasts. Subgroup analyses by ethnicity and source of control were performed subsequently. *χ*^2^-based Q-statistic test was employed to explore statistical heterogeneity with *P* < 0.05 for statistical significance. A quantitative measure of between-study heterogeneity was also investigated using the *I*^2^ statistic, and the heterogeneity was defined as low, moderate, and high based on *I*^2^ values of 25%, 50%, and 75%, respectively.^[17]^ If significant heterogeneity existed (*P* < .05), the random-effects model was used to pool overall effects; otherwise, a fixed-effect model was selected. Sensitivity analysis was undertook by excluding each single study in turn to investigate the stability of results. Begg funnel plot and Egger test were adopted to detect any underlying publication bias in the meta-analysis. Finally, the distribution of genotypes in controls was tested for a departure from HWE using *χ*^2^ test. All statistical analyses in this study were implemented with STATA 12.0 software (Stata Corporation, College Station, TX).

## 3 Results

### 3.1 Study characteristics

**Figure 1:**
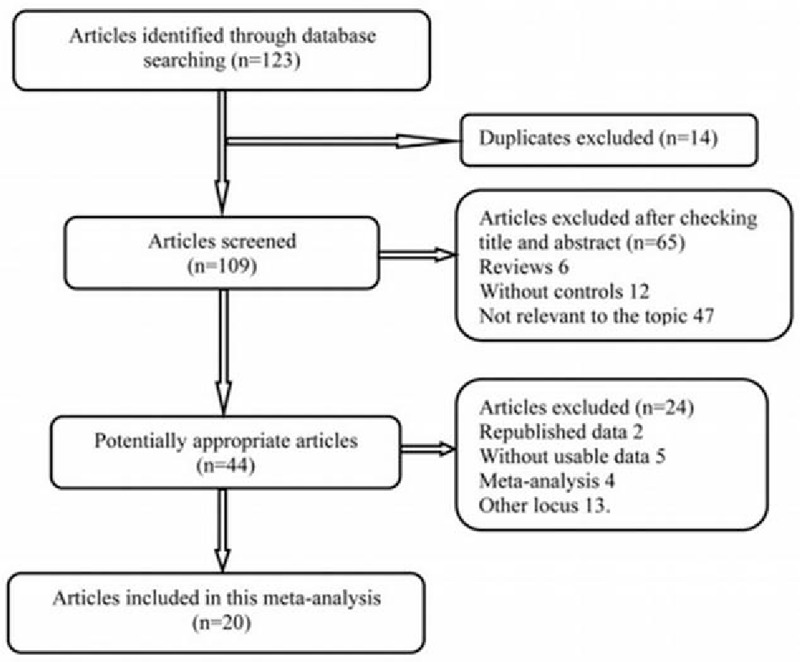
Flow diagram of article selection for the meta-analysis on the association between *SRD5A2* rs9282858 polymorphism and prostate cancer susceptibility.

In accordance with search strategy, 123 articles were searched from electronic databases originally. Apart from 14 duplicates, another 65 records were also deleted because of reviews (n = 6), without controls (n = 12) and irrelevant to the topic (n = 47), and 24 articles were removed because of republished data (n = 2), unusable data (n = 5), meta-analysis (n = 4), and not involving rs9282858 polymorphism (n = 13). Ultimately, 20 articles^[13–15,18–34]^ incorporating 30 case-control studies were included in the meta-analysis, involving a total of 7300 cases and 7952 controls. The selection process and characteristics of eligible studies are displayed in Figure 1 and Table 1, respectively. The genotype distributions of the controls were consistent with HWE in all included studies (*P* > .05).

**Table 1:**
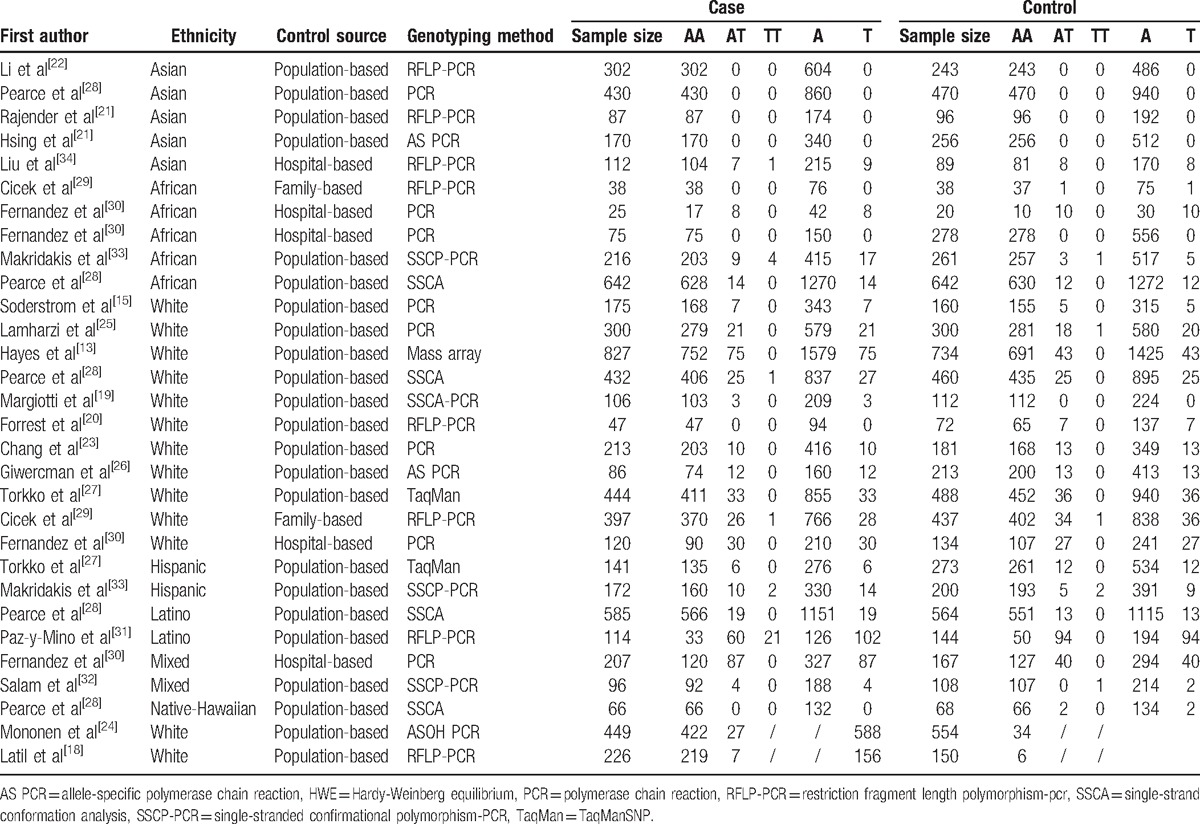
Principal characteristics of the studies included in the meta-analysis.

### 3.2 Meta-analysis results

**Figure 2:**
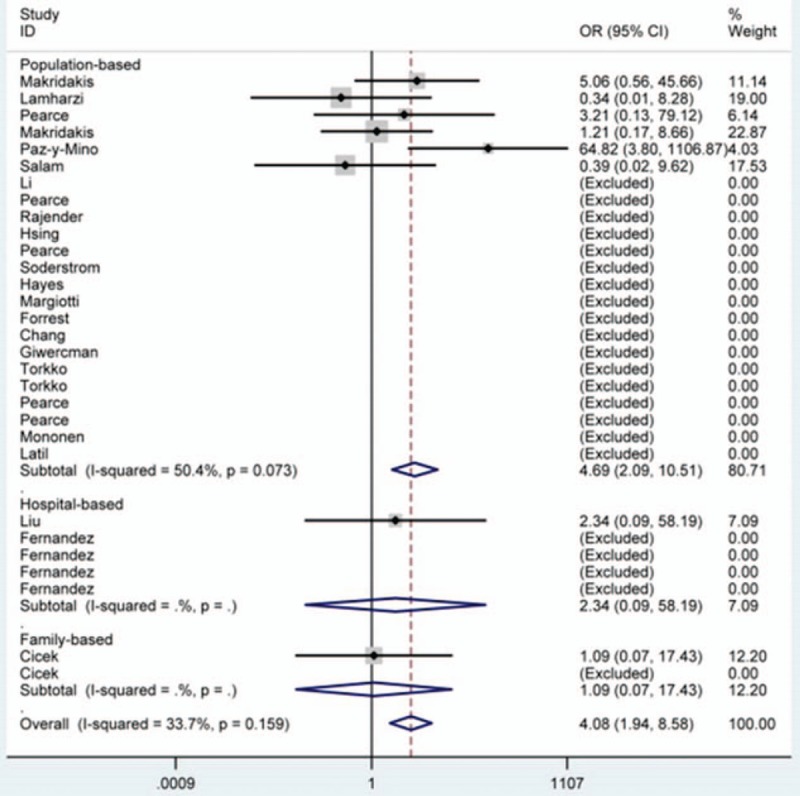
Forest plot of prostate cancer susceptibility associated with *SRD5A2* rs9282858 polymorphism under TT vs. AA model after stratification analysis by control source. CI = confidence interval, OR = odds ratio.

**Figure 3:**
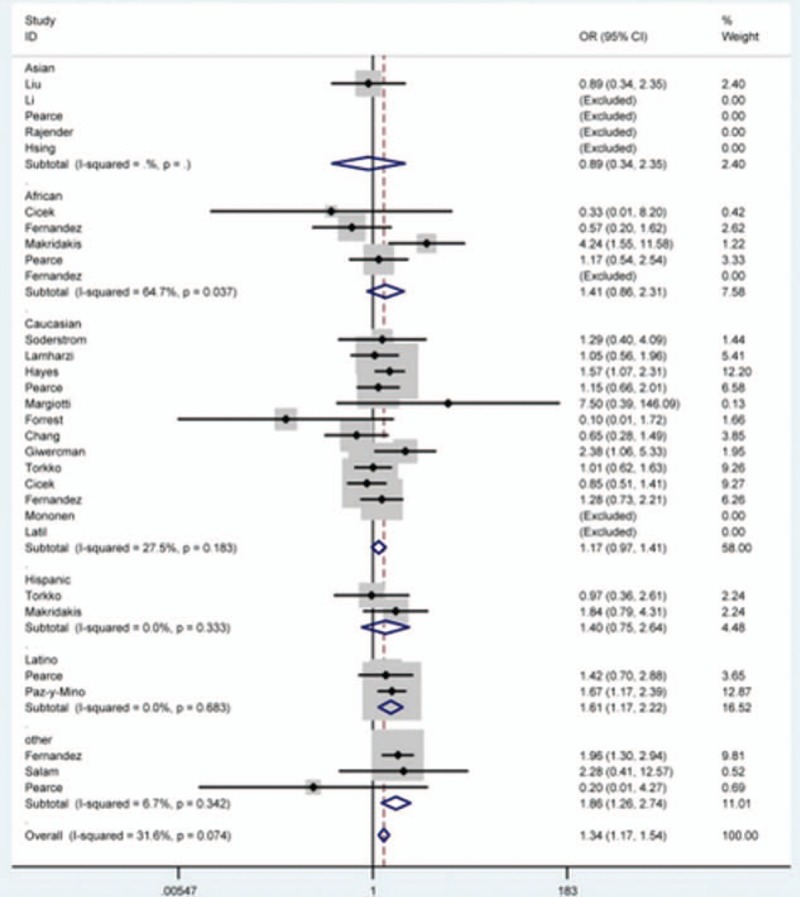
Forest plot of prostate cancer susceptibility associated with *SRD5A2* rs9282858 polymorphism under allele T vs. allele A model after stratification analysis by ethnicity. CI = confidence interval, OR = odds ratio.

Table 2 presents the main results of this meta-analysis. Overall, we found that there was a statistically significant relationship between *SRD5A2* rs9282858 polymorphism and increased PCa susceptibility (TT vs. AA: OR = 4.08, 95% CI = 1.94–8.58 [Fig. 2]; TT + AT vs. AA: OR = 1.28, 95% CI = 1.11–1.47; TT vs. AA + AT: OR = 4.44, 95% CI = 2.12–9.27; allele T vs. allele A: OR = 1.34, 95% CI = 1.17–1.54 [Fig. 3]).

**Table 2:**
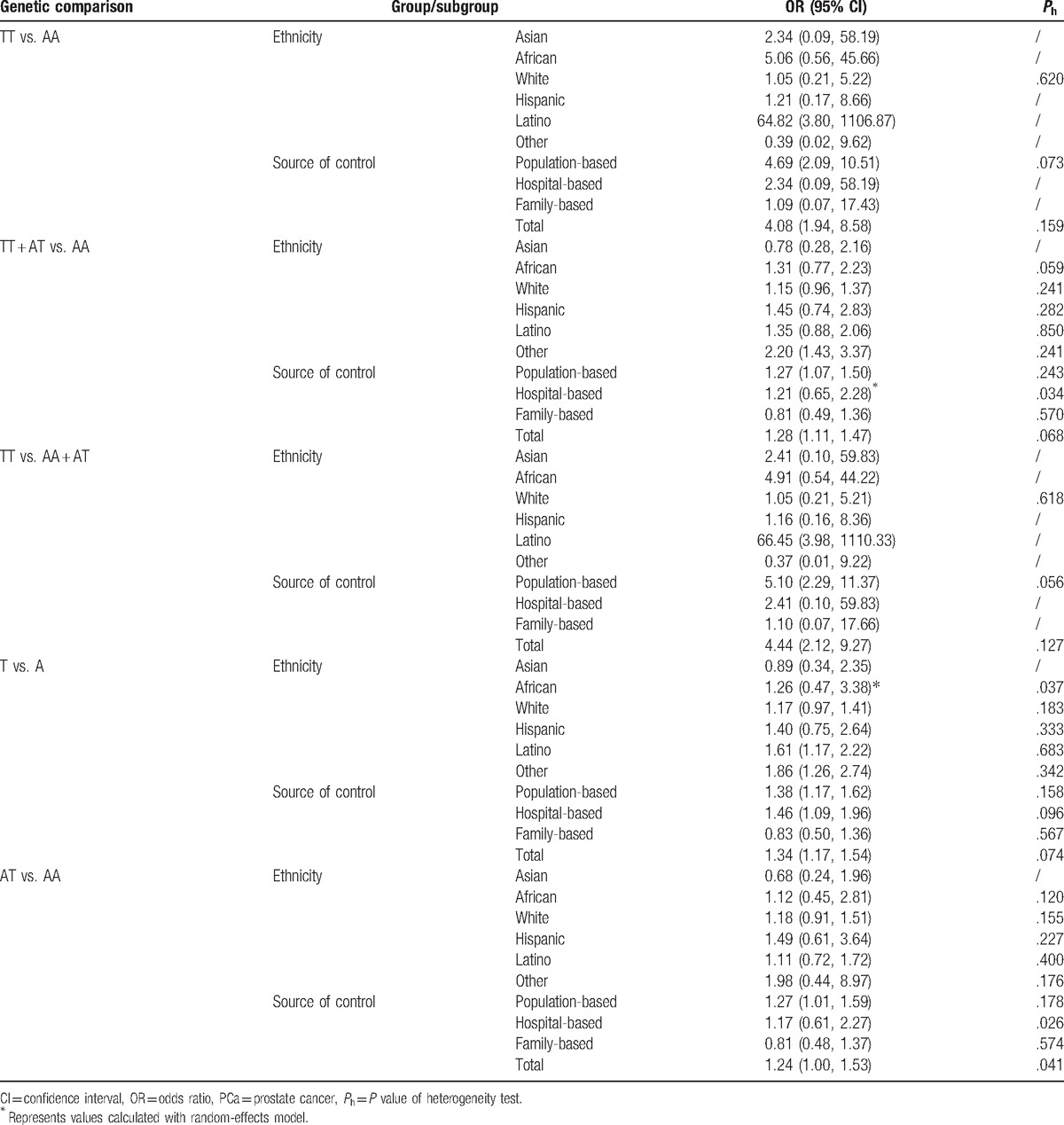
*SRD5A2* rs9282858 polymorphism and the susceptibility of PCa.

After subgroup analysis based on ethnicity, the susceptibility of PCa was notably enhanced in Latinos (TT vs. AA: OR = 64.82, 95% CI = 3.80–1106.87; TT vs. AA + AT: OR = 66.45, 95% CI = 3.98–1110.33; allele T vs. allele A: OR = 1.61, 95% CI = 1.17–2.22 [Fig. 3]) and in other-ethnicity (TT + AT vs. AA: OR = 2.20, 95% CI = 1.43–3.37; allele T vs. allele A: OR = 1.86, 95% CI = 1.26–2.74 [Fig. 3]) groups.

What is more, after stratified analysis by source of control, similar results were observed in population-based (TT vs. AA: OR = 4.69, 95% CI = 2.09–10.51 [Fig. 2]; TT + AT vs. AA: OR = 1.27, 95% CI = 1.07–1.50; TT vs. AA + AT: OR = 5.10, 95% CI = 2.29–11.37; allele T vs. allele A: OR = 1.38, 95% CI = 1.17–1.62; AT vs. AA: OR = 1.27, 95% CI = 1.01–1.59) and hospital-based (allele T vs. allele A: OR = 1.46, 95% CI = 1.09–1.96) subgroups as well.

### 3.3 Heterogeneity test

As shown in Table 2, there was significant heterogeneity under AT versus AA model (*P* = .041), so we chose the random-effects model to calculate pooled results. Additionally, after stratification analysis by control source, we observed that studies enrolling controls from hospitals might be the source of significant heterogeneity.

Meanwhile, as no substantial heterogeneity was found under TT versus AA (*P* = .159), TT + AT versus AA (*P* = .068), TT versus AA + AT (*P* = .127), and allele T versus allele A (*P* = .074) comparisons, a fixed-effect model was therefore applied.

### 3.4 Sensitivity analysis and publication bias

Through removing one single included study each time, sensitivity analysis was completed, and no substantial change was observed during the whole course, reflecting that the results of this meta-analysis were stable and credible.

**Figure 4:**
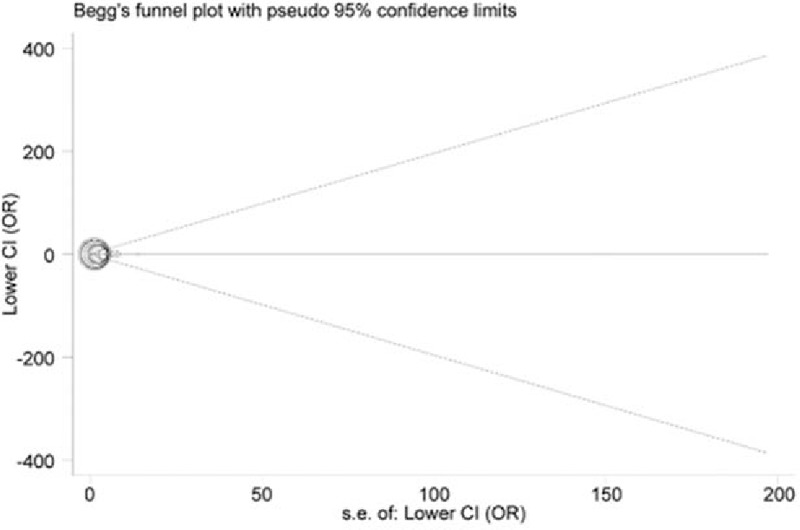
Begg funnel plot for publication bias under the model AT vs. AA.

As for publication bias, the shapes of all funnel plots seemed symmetrical (Fig. 4), and statistical values from Egger test also supported these results (TT vs. AA: *P* = .356; TT + AT vs. AA: *P* = .228; TT vs. AA + AT: *P* = .353; allele T vs. allele A: *P* = .282; AT vs. AA: *P* = .253), demonstrating that there was no significant publication bias.

## 4 Discussion

PCa is one of the common diseases in older men, frequently occurring in western countries in particular.^[1]^ Up to now, the pathogenesis of PCa has not been identified utterly, but a few risk factors have been recognized, including heredity, high-fat diet, low intake of isoflavone, and excessive intake of pickled meats.^[35–38]^ Recently, reduce of copy number in *AR* gene CAG repeat sequences is also proposed to possibly conduce to the occurrence of PCa.^[7]^

Steroid 5α-reductase is a membrane protein in microsomal membrane and nuclear membrane principally, consisting of SRD5A1 and SRD5A2. The gene *SRD5A2* coding for the latter is located at chromosome 2p23, containing 5 exons and encoding 254 oxyacids. Investigations have displayed that *SRD5A2* SNPs may change the activity of 5α-reductase, so the rs9282858 polymorphism, among others, has attracted increasing attention from scholars for the past few decades. So far, numerous studies have been conducted on the relationship between *SRD5A2* rs9282858 polymorphism and PCa susceptibility, but the conclusions are not unanimous. A few meta-analyses^[39–42]^ were also conducted to figure out the influence of *SRD5A2* rs9282858 polymorphism on PCa susceptibility. However, because of different inclusive criteria and uneven sample sizes, these reports presented different conclusions. Among them, the most recent one^[42]^ was conducted in 2012 based on 17 case-control studies, including 13 in whites, 3 in Asians, and 1 in Africans. Thus, it is of great significance to perform a more comprehensive meta-analysis with the updated publications.

This meta-analysis, including 30 independent case-control studies with 7300 cases and 7952 controls, was strictly implemented from literature search to information abstract to data syntheses. Compared to the previous studies, this meta-analysis had several strengths. Most importantly, our relatively larger sample size provided the analysis results with strong statistical power, and subgroup analyses by ethnicity and control source also facilitated a more detailed interpretation of the impact of *SRD5A2* rs9282858 polymorphism on PCa in specific populations. In this study, our findings revealed that *SRD5A2* rs9282858 polymorphism increased PCa susceptibility under all but one comparison in total analysis. Furthermore, after stratified analyses by ethnicity and source of control, a similar association was also found in Latinos, other-ethnicity, population-based, and hospital-based subgroups under corresponding genetic models. Although in terms of Latino groups the data are distinguished between the studies of Pearce et al^[28]^ and Paz-y-Mino et al^[31]^, no substantial heterogeneity was found between the studies (Fig. 3). Since the selection criteria for study subjects varied among the studies, basic characteristics such as age period, pathological tumor stage, and lifestyles might be statistically different. Besides, the sample sizes in the studies were uneven and various genotype detection methods may also contribute to biased conclusions. Research by Uemura et al^[43]^ indicated that steroid 5α-reductase is a culture medium of 11-deoxycorticosterone, and that it might contribute to the proliferation and differentiation of PCa cells via regulating AR pathway. The rs9282858 (A49T) is a common polymorphism site in the *SRD5A2* gene, which results in an alanine residue at codon 49 being replaced with threonine.^[33]^ Furthermore, in vitro experiments uncovered that the presence of rs9282858 missense mutation could increase the activity of steroid 5α-reductase by 5 times,^[28,33]^ thereby affecting the onset risk of PCa.

Despite certain advantages, some insurmountable limitations in this meta-analysis should be addressed. First, only published studies were included, and publication bias might have been generated, even though it was not detected in relevant tests. Second, although no language restrictions were imposed on literature search strategy, only articles written in English or Chinese language were finally reviewed. This is because of the language ability and the right to use databases of our team, and also might result in some bias. Third, the sample sizes of subgroup analyses were relatively small, which might affect the accuracy of our results and even the subsequent conclusions. Fourth, since meta-analysis is secondary analysis,^[44]^ we could not evaluate the possible impacts of gene-gene and gene-environment interactions on the pathogenesis of PCa in this work because of the lack of original information.

Taken together, the present meta-analysis offers additionally strong evidence for the association between the rs9282858 polymorphism in *SRD5A2* and increased PCa susceptibility. It provides an important theoretical basis to reveal the rs9282858 polymorphism and the biological mechanism of developing PCa, which may be helpful to predict the occurrence of PCa. Importantly, more attention should be paid to the roles of polymorphisms in clinical aggressiveness and therapeutic response in future work. Considering the above-mentioned restrictions, more larger-scale and well-designed studies based on multiple ethnic populations are needed to confirm our results in future.
